# Does crural repair with biosynthetic mesh improve outcomes of revisional surgery for recurrent hiatal hernia?

**DOI:** 10.1007/s10029-024-03023-x

**Published:** 2024-03-29

**Authors:** T. Panici Tonucci, A. Aiolfi, D. Bona, L. Bonavina

**Affiliations:** 1 Division of General and Foregut Surgery, Department of Biomedical Sciences for Health, IRCCS Policlinico San Donato, University of Milan, Via Piercandido Decembrio 19/A, 20137 Milan, Italy; 2 Division of General Surgery, Department of Biomedical Sciences for Health, I.R.C.C.S. Ospedale Galeazzi – Sant’Ambrogio, University of Milan, Milan, Italy

**Keywords:** Hiatus hernia, Recurrent hiatal hernia, Crural repair, Failed fundoplication, Biosynthetic mesh, GERD-HRQL score

## Abstract

**Background:**

Laparoscopic revisional surgery for recurrent hiatal hernia (HH) is technically demanding. Re-recurrences are common and esophageal hiatus mesh reinforcement might improve durability of the repair, thus minimizing the risk of re-herniation.

**Purpose:**

Assess safety and effectiveness of simple suture repair (no mesh group) vs. crural augmentation with a biosynthetic absorbable mesh (mesh group) in patients with recurrent HH.

**Methods:**

Observational retrospective study from September 2012 to December 2022. Only patients undergoing redo surgery for previous failures of hiatal hernia repair were enrolled. Surgical failure was defined as symptomatic recurrent HH with > 2 cm of gastric tissue above the diaphragmatic impression at upper gastrointestinal endoscopy and/or swallow study. Gastro-Esophageal Reflux Disease Health-Related Quality of Life (GERD-HRQL) and Short Form-36 (SF-36) questionnaires were used to assess and preoperative and postoperative symptoms and quality of life.

**Results:**

One hundred four patients were included. Overall, 60 patients (57.7%) underwent mesh-reinforced cruroplasty, whereas 44 (42.3%) underwent simple suture cruroplasty. Mesh and no mesh groups had similar baseline demographics, symptoms, prevalence of esophagitis and Barrett’s esophagus, and HH size. A composite crural repair was most commonly performed in the mesh group (38.3% vs. 20.5%; *p* = 0.07). In addition to cruroplasty, most patients (91%) underwent a Toupet fundoplication. The 90-day postoperative complication rate was 8.6%, and there was no mortality. Recurrent HH was diagnosed in 21 patients (20.2%) with a clinical trend toward reduced incidence in the mesh group (16.7% vs. 25%; *p* = 0.06). Compared to baseline, there was a statistically significant improvement of median GERD-HRQL score (*p* < 0.01) and all SF-36 items (*p* < 0.01).

**Conclusions:**

Laparoscopic revisional surgery for recurrent HH is safe and effective. Selective use of biosynthetic mesh may protect from early recurrence and has the potential to reduce re-herniation in the long-term.

**Supplementary Information:**

The online version contains supplementary material available at 10.1007/s10029-024-03023-x.

## Introduction

Anatomic and/or symptomatic recurrence is the Achilles’ heel of hiatus hernia (HH) repair. Depending on definitions, relapse rates up to 57% have been reported [1–3]. Etiology and risk factors for recurrence are multiple and still not completely understood [4, 5]. Several techniques devised to protect or release tension on the hiatal repair (posterior fundoplication, crural mesh, falciform ligament flap, relaxing diaphragmatic incisions), to anchor the esophagogastric junction/stomach in the abdomen (posterior gastropexy, anterior gastropexy or gastrostomy), or to lengthen the esophagus (wedge resection of the gastric fundus or truncal vagotomy) have been applied in an attempt to reduce the risk of HH recurrence [6].

Revisional operations for recurrent HH are challenging. The current standard of care in patients with recurrent symptomatic HH is laparoscopic revisional cruroplasty and fundoplication, which is technically demanding and should be reserved to patients with refractory symptoms [7]. Despite revisional surgery is associated with increased postoperative morbidity compared to the primary operation, previous studies have reported satisfactory short-term outcomes with up to 80% symptoms resolution and improved quality of life [8, 9]. However, re-recurrences are still common and there is a lot of ongoing research to solve the dilemma of the root cause of re-herniation and to determine whether it is more important to reconstruct and reinforce the hiatus, to anchor the esophagogastric junction below the diaphragm, or to lengthen the esophagus. Reconstruction of the esophageal hiatus has been the focus of surgeons for decades [10–12]. Over the past two decades, the use of biosynthetic mesh has been proposed to improve durability of the repair while reducing the risk of visceral erosion and severe complications associated with non-absorbable meshes [13–15].

The aim of this study was to examine our 10-year experience with recurrent HH repairs comparing safety and efficacy of simple suture crural repair vs. crural augmentation with a biosynthetic absorbable mesh.

## Materials and methods

This a retrospective, comparative study with data extracted from a prospectively maintained database. Patients were enrolled in two academic institutions from September 2012 to December 2022. Patients who underwent elective laparoscopic crural repair for symptomatic HH recurrence were included. The institutional review board (IRB) approved the study (HR#109-2023) and all patients signed a written informed consent.

### Inclusion and exclusion criteria

Inclusion criteria (a) adult patients (≥ 18 years old), (b) symptomatic HH recurrence managed with Phasix-ST® mesh reinforced cruroplasty (mesh group) vs. simple suture cruroplasty (no mesh group) and fundoplication, (c) elective surgery, (d) patients who completed the pre- and postoperative instrumental assessment and the gastroesophageal reflux disease health-related quality of life (GERD-HRQL) and short-form 36 (SF-36) questionnaires (e) patients with a minimum 12-month follow-up. Exclusion criteria (a) previous Heller myotomy or esophagogastric resection (b) emergency repair, (c) patients with < 12-month follow-up, (d) patients managed with mesh other than Phasix-ST®.

### Data collection

Baseline demographics and patient characteristics, such as age, sex, body mass index (BMI) (kg/m^2^), American Society of Anesthesiologists (ASA) physical status, comorbidities, HH size, operative data, and short-term surgical outcomes (90-day morbidity and mortality), were collected. Preoperative evaluation routinely included upper gastrointestinal endoscopy and barium swallow study. Perioperative complications were defined in accordance with the Clavien–Dindo classification [16].

### Surgical technique

**Fig. 1:**
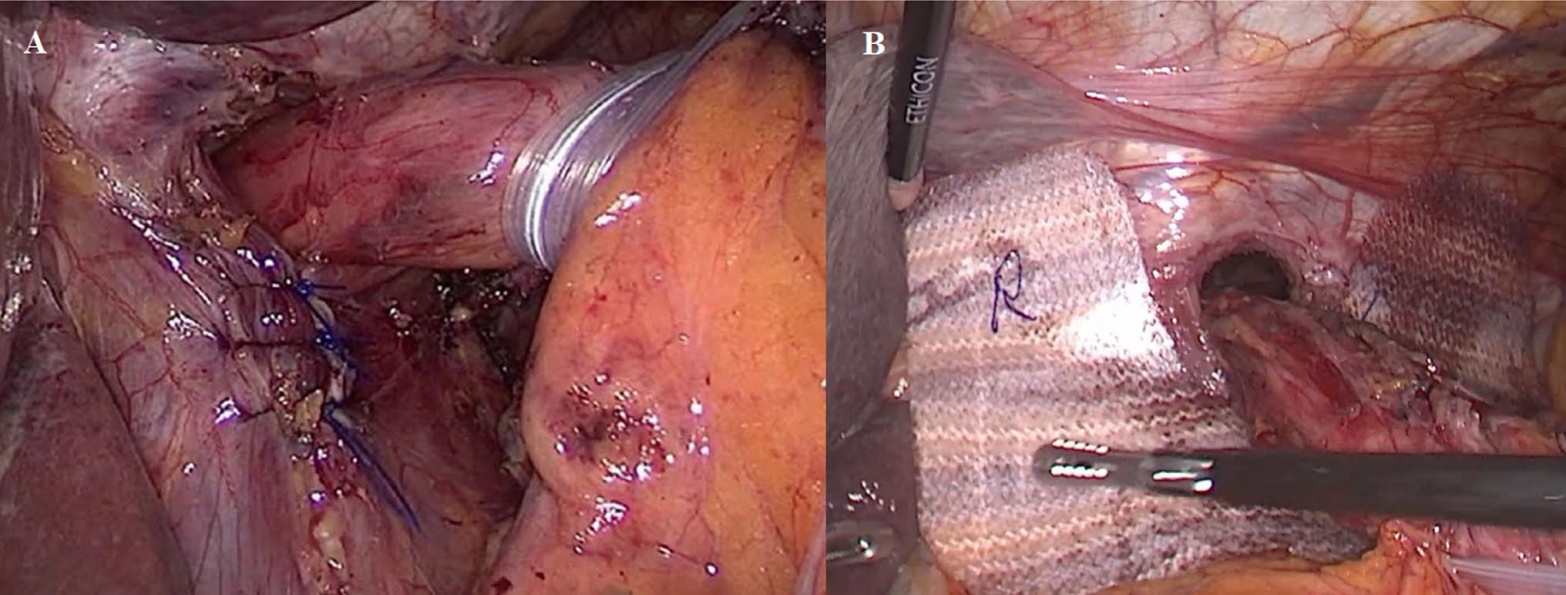
**A** The hiatus is closed with interrupted non-absorbable sutures. **B** The U-shape Phasix-ST mesh is placed at the hiatus to reinforce the suture and fixed in place with absorbable braided stitches

All revisional surgical procedures were performed laparoscopically. After taking down adhesions from previous surgery and careful dissection around the hiatus, all hernia contents and hernia sac were reduced, the distal esophagus was encircled with a Penrose drain, and traction was applied to obtain a 3–5 cm tension-free intra-abdominal segment. If present, the previous fundoplication was taken down until the angle of His was clearly identified. The posterior hiatoplasty was then performed with interrupted non-resorbable sutures (Prolene® 2.0-Ethicon) tied with extracorporeal knots. A composite dorsal–ventral repair with stitches including the apex of the left crus and the central tendon of the diaphragm was performed in selected patients with larger hiatal defect to avoid excessive angulation and narrowing of the esophagus. Care was taken to reduce the pneumoperitoneum from 13 to 6 mmHg during the hiatoplasty [17]. The decision to place or not a mesh for crural reinforcement was initially dependent on the operating surgeon preference and/or subjective assessment indicating weak crura or tissue tearing from sutures. From January 2021, the procedure was chosen according to a standardized the patient treatment algorithm [9, 10]. In case of simple suture cruroplasty (no mesh), the reconstruction of the diaphragmatic pillars was performed with interrupted non-resorbable sutures (Prolene® 2.0-Ethicon) tied with an extracorporeal knot. In case of mesh reinforced cruroplasty, after mediastinal dissection and cruroplasty with sutures, a 7 × 10 cm Phasix-ST® mesh made of poly-4-hydroxybutyrate (P4HB) with a hydrogel barrier on one side was shaped with ‘‘U’’ configuration and fixed over the approximated hiatus surface with at least two interrupted absorbable sutures (Fig. 1). Finally, after division of the first 2–3 short gastric vessels, a posterior fundoplication was fashioned according to the previously described critical view of safety approach [18]. To prevent postoperative nausea and vomiting, an 8 mg bolus of dexamethasone was routinely administered during the surgical procedure. The nasogastric tube was removed at the end of the operation. A chest film and a gastrografin swallow study were performed on postoperative day 1 to check the correct position of the esophagogastric junction. A soft diet was then allowed, and patients were discharged home with proper diet instructions.

### Follow-up

Outpatient follow-up visits were scheduled at 1, 6, and 12 months after the operation, and then yearly. Endoscopic and radiological (upper gastrointestinal swallow study and computed tomography) findings were collected. Barium swallow study and/or upper gastrointestinal endoscopy were performed between 6 and 12 months after surgery and repeated every year or at any time the patient complained of symptoms. Disease-specific Gastro-Esophageal Reflux Disease Health-Related Quality of Life (GERD-HRQL) and generic Short Form-36 (SF-36) were used to assess patients’ quality of life at baseline and during follow-up [19].

### Outcomes and definitions

The primary aim was to compare the safety and efficacy of revisional surgery for symptomatic HH recurrence and compare data between mesh and no mesh. Secondary outcomes were short-term (90-day) surgical complications, changes in esophageal symptoms, and patient-related quality of life assessed with GERD-HRQL and SF-36. Surgical failure was defined as symptomatic recurrent HH with > 2 cm of gastric tissue above the diaphragmatic impression at upper endoscopy and/or swallow study.

### Statistical analysis

Continuous data are presented as mean and standard deviation or median and IQR. Categorical variables are shown as numbers and percentages. Wilcoxon signed-rank for paired data was performed as appropriate. Two-sided *p* values were computed. Statistical significance was considered when *p* value was equal or less than 0.05. Confidence interval was set at 95% confidence level. Recurrence-free probability was estimated with the Kaplan–Meier method. The hazard function was estimated using non-parametric smoothing method. All analyses were carried out using R version 3.2.2 software [20].

## Results

**Fig. 2:**
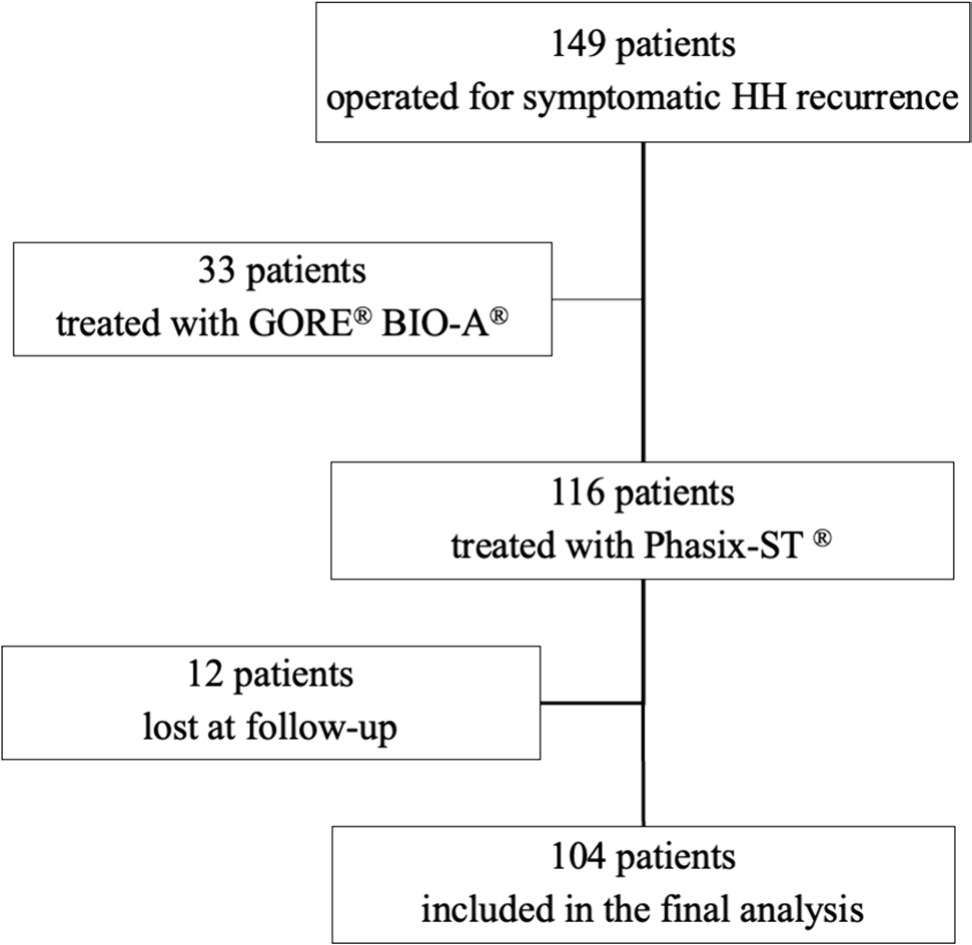
Flow chart of the study population including patient recruitment, exclusion criteria, and lost at follow-up

During the study period, 149 patients were treated for HH recurrence. One hundred four patients were included in the final analysis (Fig. 2). Overall 60 (57.7%) underwent mesh-reinforced cruroplasty whereas 44 (42.3%) underwent simple suture cruroplasty. Demographics of the patient population are shown in Table 1. Age, gender, BMI (kg/m^2^), ASA score, comorbidities, and smoke status were comparable between groups. Heartburn (79.8%), dysphagia (40.4%), regurgitation (28.8%), and chest pain (26.9%) were the most common typical esophageal symptoms, while 27% of patients complained atypical symptoms. Esophagitis or Barrett’s esophagus was diagnosed in 35.6% and 11.5% of patients, respectively. Symptom’s duration, length of PPI therapy, and daily PPI intake were also comparable. Preoperative endoscopic assessment showed a trend toward a larger HH size in the mesh group (4.1 vs. 3.4; *p* = 0.07). Overall, 5 patients (4.8%) had previous crural mesh repair and 91.3% had a Nissen fundoplication. The median preoperative GERD-HRQL score was 23 (range 8–44).

**Table 1:** Demographic and preoperative characteristics of the patient population

	Mesh group (*n* = 60)	No Mesh group (*n* = 44)	*p *value
Sex, female, *n* (%)	38 (63.3)	26 (59.1)	0.19
Age, yrs, median (IQR)	58 (51–68)	53 (45–65)	0.09
BMI, median (IQR)	25.6 (23.3–27.1)	25.1 (22.4–27.9)	0.79
ASA score > 2 *n* (%)	10 (16.7)	7 (15.9)	0.65
Active smoker, *n* (%)	7 (11.7)	5 (11.3)	0.72
Comorbidities, *n* (%)			
CAD	9 (15)	6 (13.6)	0.28
COPD	8 (13.3)	6 (13.6)	0.91
Hypertension	17 (28.3)	11 (25)	0.37
Diabetes	5 (8.3)	4 (9.1)	0.17
Kyphoscoliosis	11 (18.3)	10 (22.7)	0.53
Anxious-depressive syndrome	13 (21.6)	11 (25)	0.08
Symptoms, *n* (%)			
Heartburn	48 (80)	35 (79.5)	0.48
Dysphagia	25 (41.7)	17 (38.6)	0.33
Regurgitation	18 (30)	12 (27.3)	0.16
Thoracic pain	16 (26.7)	12 (27.2)	0.51
Dyspnea	8 (13.3)	5 (11.4)	0.74
Anemia/previous blood transfusion	3 (5)	2 (4.5)	0.37
Symptom duration, mos, median (IQR)	26.5 (12–96)	35 (24–84)	0.29
PPI therapy, *n* (%)	46 (76.7)	32 (72.7)	0.49
PPI therapy duration, mos, median (IQR)	72 (24–120)	70 (48–108)	0.39
Hiatus hernia, cm, median (IQR)	4.1 (2–6)	3.4 (2–5)	0.07
GERD–HRQL score, median (IQR)	23 (8–41)	21 (6–40)	0.81
Esophagitis (grade A–D), *n* (%)	22 (36.7)	15 (34.1)	0.29
Barrett esophagus, *n* (%)	7 (11.7)	5 (11.4)	0.36
Previous operation, *n* (%)			
*n* = 1	49 (81.7)	39 (88.6)	0.17
*n* = 2	11 (18.3)	5 (10.4)	
With mesh	4 (6.7)	1 (2.3)	0.37
Previous fundoplication, *n* (%)			
Nissen	55 (91.7)	40 (91)	0.89
Toupet	5 (8.3)	4 (9)	
Time from previous surgery, mos, median (IQR)	39 (13–68)	46 (8–81)	0.65

Values are expressed as median (interquartile range—IQR) or numbers (%)

*BMI* Body Mass Index, *ASA* American Society of Anesthesiologists classification, *CAD* coronary artery disease, *COPD* chronic obstructive pulmonary disease, *PPI* proton pump inhibitors, *mos* months

All operations were completed laparoscopically. The median operative time was 122 min (range 54–210) (Table 2). No intraoperative complications occurred, and intraoperative blood loss was negligible; the posterior vagal trunk was identified and preserved in 101 patients (97%), and there were no conversions to open procedures. The composite posterolateral cruroplasty was most commonly performed in patients that underwent mesh repair (38.3% vs. 20.5%; *p* = 0.07). Ninety-one percent of patients underwent a Toupet fundoplication. The median postoperative hospital stay was 3 days (IQR 1–8). The 90-day postoperative complication rate was 8.6% (*n* = 9). The distribution of complications according to the Clavien classification was the following: pleural effusion requiring drainage (IIIa) (*n* = 3), gastroparesis requiring endoscopic dilation (IIIa) (*n* = 3), hydropneumothorax and pleural empyema (grade IIIb) (*n* = 1), thromboembolism (grade IV) (*n* = 1), and esophageal perforation managed endoscopically (grade IV) (*n* = 1). There was no mortality.

**Table 2:** Intraoperative variables according to the mesh vs. no mesh buttressing

	Mesh group (*n* = 60)	No Mesh group (*n* = 44)	*p *value
Hiatoplasty, *n* (%)			
Posterior	37 (61.7)	35 (79.5)	0.07
Composite	23 (38.3)	9 (20.5)	
Numbers of hiatoplasty stitches, median (IQR)			
Posterior	3 (3–4)	3 (2–4)	0.16
Left lateral	1 (1–2)	2 (2–3)	0.25
Fundoplication, *n* (%)			
Toupet (CVS)	56 (93.3)	39 (88.6)	0.24
Nissen	1 (1.7)	3 (6.8)	0.21
Lortat-Jacob	1 (1.7)	1 (2.3)	0.32
Dor	2 (3.3)	1 (2.3)	0.54
Falciform ligament flap, *n* (%)	2 (3.3)	7 (15.9)	0.03
Wedged Collis gastroplasty, *n* (%)	2 (3.3)	2 (4.5)	0.32
OT, min, median (IQR)	202 (142–287)	189 (128–298)	0.19
90-day morbidity, *n* (%)	5 (8.3)	4 (9.1)	0.91

Values are expressed as median (IQR—interquartile range) or numbers (%)

*CVS* critical view of safety, *OT* operative time, *min* minutes

**Fig. 3:**
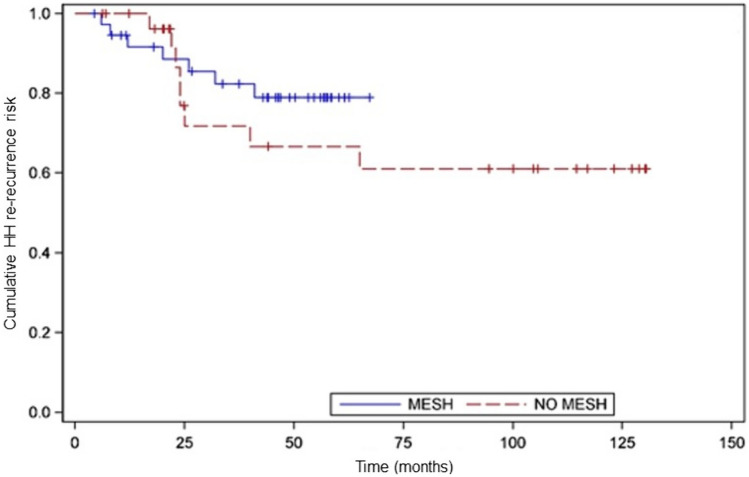
Kaplan–Meier survival curve in patients treated with mesh (blue line) and no mesh (red line). *X* axis shows time expressed in months. *Y* axis shows the cumulative risk for HH re-recurrence

The median postoperative follow-up time was shorter (p = 0.039) in the mesh group (55 months, IQR 6–64) compared to the no mesh group (102 months, IQR 8–128) (Table 3). Overall, recurrent HH was diagnosed in 21 patients (20.2%) and redo surgery was necessary in five patients. Despite the lack of statistical significance, there was a clinical trend toward reduced hernia recurrence in patients who underwent mesh repair (16.7% vs. 25%; *p* = 0.06) (Fig. 3). Specifically, the recurrence-free probability for mesh vs. no mesh repair at 24 months was 0.96 (95% CI 0.87–0.98) vs. 0.93 (95% CI 0.76–0.97), 0.84 (95% CI 0.67–0.91) vs. 0.75 (95% CI 0.68–0.86) at 36 months, and 0.81 (95% CI 0.65–0.89) vs. 0.69 (95% CI 0.62–0.79) at 48 months, respectively. The timeline analysis showed a comparable median time of HH recurrence between mesh (20 mos, IQR 8–32) and no mesh (24 months; IQR 6–36) patients (*p* = 0.24). At the last follow-up, 80% of the patients were off PPI, with 20% taking daily or occasional PPI for residual symptoms. The median GERD-HRQL was significantly improved compared to baseline in mesh (*p* = 0.012) and no mesh patients (*p* = 0.023). All SF-36 items (Supplementary Table 1) were significantly improved (*p* < 0.001) compared to baseline.

**Table 3:** Follow-up variables according to the mesh vs. no mesh buttressing

	Mesh group (*n* = 60)	No Mesh group (*n* = 44)	*p *value
Follow-up, mos, median (IQR)	55 (6–64)	102 (18–128)	0.02
PPI off, *n* (%)	49 (81.7)	34 (77.3)	0.21
GERD-HRQL score, median (IQR)	7 (3–13)	8 (4–15)	0.51
Recurrent HH, *n* (%)	10 (16.7)	11 (25)	0.06
Redo surgery for HH re-recurrence, *n* (%)	2 (3.3)	3 (8.8)	0.08

Values are expressed as median (IQR—interquartile range) or numbers (%)

*PPI* proton pump inhibitors therapy, *mos* months, *HH* hiatus hernia

## Discussion

The present study demonstrates the safety and the efficacy of laparoscopic repair of recurrent HH with either simple suture or mesh reinforced cruroplasty over a median follow-up > 5 years. Also, there was a clinical trend toward reduced HH re-herniation in patients who received  biosynthetic mesh (Phasix-ST®) augmentation.

Reoperations for recurrent HH are technically challenging and associated with higher postoperative morbidity and conversions to open surgery [21, 22]. Multiple factors are potentially associated with the difficulties encountered during these revisional procedures, such as distorted anatomy, dense adhesions, tissue scarring, and progressive deterioration of esophagogastric physiology. In our series, none of the patients required conversion to open procedure and intraoperative complications were negligible. Furthermore, the postoperative complication rate was lower (8.6%) compared to previous studies reporting up to 29% complication rates [23, 24]. Notably, we performed a composite crural repair in both patient groups, and the Toupet fundoplication was our preferred surgical technique to minimize the risk of postoperative dysphagia and gas bloat syndrome [25].

Laparoscopic repair with mesh-reinforced cruroplasty has become an increasingly common surgical approach in patients with primary HH [26, 27]. Recent systematic reviews and meta-analyses show conflicting results in terms of recurrence prevention due to heterogeneity [28]. Furthermore, the safety and the efficacy of mesh augmentation in the setting of redo surgery for HH recurrence are even more indeterminate because evidence is scarce. Complications related to non-absorbable mesh, including esophageal strictures, abscess or fistula formation, and full-thickness erosions related to the process of mesh scarring, have been extensively reported after both primary repair and redo surgery [29]. In our patient population, no mesh-related complications occurred and none of the patient’s required reoperation for related complications. This may be attributable to a combination of both P4HB and Sepra Technology (ST) coating of the Phasix-ST® mesh. This barrier of sodium hyaluronate and carboxymethylcellulose applied to one side of the mesh seems important to minimize the risk of visceral adhesions [30, 31].

The overall hernia re-recurrence rate in this study was 20.2%. This is comparable to previous studies reporting data from redo surgery after failed primary HH repair [32–35]. Also, a recent meta-analysis reported a weighted pooled proportion of recurrence across the studies of 10.7% (95% CI 7.7–14.1%) [36]. In our experience, only five patients required further surgical revision whereas the majority remained asymptomatic on PPI medications. There was a clinical trend toward reduced recurrence with mesh-reinforced cruroplasty compared to simple suture cruroplasty (16.7% vs. 25%). The lack of statistical significance may be due to the limited sample size in our study. The majority of recurrences were diagnosed after 20 months from the operation, indicating that the estimated time of absorption of the Phasix-ST mesh should provide enough time for hiatus reinforcement and protection from early failures [37–40]. Although cruroplasty with mesh may have a role in reducing postoperative re-recurrences after failed primary HH repair, confirmation on large and well-conducted observational studies or randomized trials is warranted [41].

Patient-reported outcomes including symptoms control and quality of life parameters are important indicators of success. Quality of life in patients with recurrent HH may be severely disrupted to the point of affecting everyday activities, social functioning, and mental health [42–44]. We found a noteworthy improvement (> 50% from baseline) in GERD-HRQL scores in both patient groups compared to baseline. Similarly, the generic SF-36 questionnaires showed an improvement in both physical and mental component summaries with improvement of physical limitations (physical functioning and role-physical), suffered pain (bodily pain), self-health perception (general health), sense of vigor (vitality), ability to participate in social activities (social functioning), and level of depression (mental health). These data are in line with a recent systematic review [45].

We acknowledge some limitations related to the retrospective design of this study. Despite the homogeneous patient population, allocation bias should be considered while interpreting our results. Further, the limited number of included patients and recurrences did not allow a robust multivariate regression analysis to assess potential risk factors for recurrence. Therefore, this preliminary comparative analysis of efficacy of Phasix-ST® mesh reinforcement versus simple repair should be interpreted cautiously and mandates future research.

## Conclusions

Laparoscopic revisional surgery for recurrent HH is safe and effective, and is associated with substantial quality of life improvement. Selective use of biosynthetic mesh may protect from early recurrence and may be beneficial to reduce re-herniation in the very long-term follow-up.

## Supplementary Information

Below is the link to the electronic supplementary material.

Supplementary file 1 (DOCX 17 KB)

## Data Availability

Not applicable.

## References

[1] Aiolfi A, Sozzi A, Cavalli M, Bonitta G, Ogliari C, Lombardo F, Lanzaro A, Bruni PG, Campanelli G, Bona D (2022) Patient-tailored algorithm for laparoscopic cruroplasty standardization: comparison with hiatal surface area and medium-term outcomes. Langenbecks Arch Surg 407(6):2537–2545. 10.1007/s00423-022-02556-y 35585260 10.1007/s00423-022-02556-y

[2] Byrne JP, Smithers BM, Nathanson LK, Martin I, Ong HS, Gotley DC (2005) Symptomatic and functional outcome after laparoscopic reoperation for failed antireflux surgery. Br J Surg 92(8):996–1001. 10.1002/bjs.4914 15997449 10.1002/bjs.4914

[3] Lamb PJ, Myers JC, Jamieson GG, Thompson SK, Devitt PG, Watson DI (2009) Long-term outcomes of revisional surgery following laparoscopic fundoplication. Br J Surg 96(4):391–397. 10.1002/bjs.6486 19283739 10.1002/bjs.6486

[4] Lidor AO, Steele KE, Stem M et al (2015) Long-term quality of life and risk factors for recurrence after laparoscopic repair of paraesophageal hernia. JAMA Surg 150:424–431 25785415 10.1001/jamasurg.2015.25

[5] Saad AR, Velanovich V (2020) Anatomic observation of recurrent hiatal hernia: recurrence or disease progression? J Am Coll Surg 230(6):999–1007. 10.1016/j.jamcollsurg.2020.03.011 32217191 10.1016/j.jamcollsurg.2020.03.011

[6] Carrera Ceron RE, Oelschlager BK (2022) Management of recurrent paraesophageal hernia. J Laparoendosc Adv Surg Tech 32(11):1148–1155. 10.1089/lap.2022.0388 10.1089/lap.2022.0388 36161967

[7] Furnée EJ, Draaisma WA, Broeders IA, Gooszen HG (2009) Surgical reintervention after failed antireflux surgery: a systematic review of the literature. J Gastrointest Surg 13(8):1539–1549. 10.1007/s11605-009-0873-z 19347410 10.1007/s11605-009-0873-z PMC2710493

[8] Dallemagne B, Arenas Sanchez M, Francart D, Perretta S, Weerts J, Markiewicz S, Jehaes C (2011) Long-term results after laparoscopic reoperation for failed antireflux procedures. Br J Surg 98(11):1581–1587. 10.1002/bjs.7590 21710482 10.1002/bjs.7590

[9] Lovece A, Sironi A, Asti E, Milito P, Boveri S, Bonavina L (2022) Laparoscopic surgery for recurrent hiatal hernia: clinical outcomes and quality of life. J Gastrointest Surg 26:917–920 34668163 10.1007/s11605-021-05165-0

[10] Aiolfi A, Cavalli M, Saino G, Sozzi A, Bonitta G, Micheletto G, Campanelli G, Bona D (2022) Laparoscopic posterior cruroplasty: a patient tailored approach. Hernia 26(2):619–626. 10.1007/s10029-020-02188-5 32335756 10.1007/s10029-020-02188-5

[11] Granderath FA, Kamolz T, Schweiger UM, Pointner R (2003) Laparoscopic refundoplication with prosthetic hiatal closure for recurrent hiatal hernia after primary failed antireflux surgery. Arch Surg 138(8):902–907. 10.1001/archsurg.138.8.902 12912751 10.1001/archsurg.138.8.902

[12] Granderath FA, Granderath UM, Pointner R (2008) Laparoscopic revisional fundoplication with circular hiatal mesh prosthesis: the long-term results. World J Surg 32(6):999–1007. 10.1007/s00268-008-9558-0 18373118 10.1007/s00268-008-9558-0

[13] Griffith PS, Valenti V, Qurashi K et al (2008) Rejection of goretex mesh used in prosthetic cruroplasty: a case series. Int J Surg 6:106–109 18234570 10.1016/j.ijsu.2007.12.004

[14] Stadlhuber RJ, Sherif AE, Mittal SK et al (2009) Mesh complications after prosthetic reinforcement of hiatal closure: a 28-case series. Surg Endosc 23:1219–1226 19067074 10.1007/s00464-008-0205-5

[15] Spiro C, Quarmby N, Gananadha S (2020) Mesh-related complications in paraoesophageal repair: a systematic review. Surg Endosc 34:4257–4280 32556700 10.1007/s00464-020-07723-0

[16] Clavien PA, Barkun J, de Oliveira ML, Vauthey JN, Dindo D, Schulick RD, de Santibañes E, Pekolj J, Slankamenac K, Bassi C, Graf R, Vonlanthen R, Padbury R, Cameron JL, Makuuchi M (2009) The Clavien–Dindo classification of surgical complications: five-year experience. Ann Surg 250(2):187–196. 10.1097/SLA.0b013e3181b13ca2 19638912 10.1097/SLA.0b013e3181b13ca2

[17] Bradley DD, Louie BE, Farivar AS, Wilshire CL, Baik PU, Aye RW (2015) Assessment and reduction of diaphragmatic tension during hiatal hernia repair. Surg Endosc 29(4):796–804. 10.1007/s00464-014-3744-y 25055892 10.1007/s00464-014-3744-y

[18] Bona D, Aiolfi A, Asti E, Bonavina L (2020) Laparoscopic toupet fundoplication for gastroesophageal reflux disease and hiatus hernia: proposal for standardization using the “critical view” concept. Updates Surg 72(2):555–558. 10.1007/s13304-020-00732-7 32130670 10.1007/s13304-020-00732-7

[19] Velanovich V (1998) Comparison of generic (SF-36) vs. disease-specific (GERD-HRQL) quality-of-life scales for gastroesophageal reflux disease. J Gastrointest Surg 2(2):141–145. 10.1016/s1091-255x(98)80004-8 9834409 10.1016/s1091-255x(98)80004-8

[20] R Core Team (2023) R: a language and environment for statistical computing. R Foundation for Statistical Computing, Vienna, Austria. http://www.R-project.org. Accessed 30 Sept 2023

[21] Kohn GP, Price RR, DeMeester SR, Zehetner J, Muensterer OJ, Awad Z, Mittal SK, Richardson WS, Stefanidis D, Fanelli RD, SAGES Guidelines Committee (2013) Guidelines for the management of hiatal hernia. Surg Endosc 27(12):4409–4428. 10.1007/s00464-013-3173-3 24018762 10.1007/s00464-013-3173-3

[22] Antiporda M, Jackson C, Smith CD, Thomas M, Elli EF, Bowers SP (2019) Strategies for surgical remediation of the multi-fundoplication failure patient. Surg Endosc 33(5):1474–1481. 10.1007/s00464-018-6429-0 30209604 10.1007/s00464-018-6429-0

[23] Wennergren J, Levy S, Bower C, Miller M, Borman D, Davenport D, Plymale M, Scott RJ (2016) Revisional paraesophageal hernia repair outcomes compare favorably to initial operations. Surg Endosc 30(9):3854–3860. 10.1007/s00464-015-4688-6 26659229 10.1007/s00464-015-4688-6

[24] Juhasz A, Sundaram A, Hoshino M, Lee TH, Mittal SK (2012) Outcomes of surgical management of symptomatic large recurrent hiatus hernia. Surg Endosc 26(6):1501–1508. 10.1007/s00464-011-2072-8 22179460 10.1007/s00464-011-2072-8

[25] Rausa E, Ferrari D, Kelly ME, Aiolfi A, Vitellaro M, Rottoli M, Bonitta G, Bona D (2023) Efficacy of laparoscopic toupet fundoplication compared to endoscopic and surgical procedures for GERD treatment: a randomized trials network meta-analysis. Langenbecks Arch Surg 408(1):52. 10.1007/s00423-023-02774-y 36680602 10.1007/s00423-023-02774-y

[26] Gerdes S, Schoppmann SF, Bonavina L, Boyle N, Müller-Stich BP, Gutschow CA, Hiatus Hernia Delphi Collaborative Group (2023) Management of paraesophageal hiatus hernia: recommendations following a European expert Delphi consensus. Surg Endosc 37(6):4555–4565. 10.1007/s00464-023-09933-8 36849562 10.1007/s00464-023-09933-8 PMC10234895

[27] Markar SR, Menon N, Guidozzi N, Kontouli KM, Mavridis D, Andreou A, Berlth F, Bonavina L, Cushieri A, Fourie L, Gossage J, Gronnier C, Hazebroek EJ, Krishnadath S, Low DE, McCord M, Pouw RE, Watson DI, Carrano FM, Ortenzi M, Antoniou SA (2023) EAES Multidisciplinary rapid guideline: systematic review, meta-analysis, GRADE assessment and evidence-informed recommendations on the surgical management of paraesophageal hernias. Surg Endosc. 10.1007/s00464-023-10511-1 37910246 10.1007/s00464-023-10511-1

[28] Angeramo CA, Schlottmann F (2022) Laparoscopic paraesophageal hernia repair: to mesh or not to mesh. Systematic review and meta-analysis. Ann Surg 275:67–72 33843796 10.1097/SLA.0000000000004913

[29] Aiolfi A, Sozzi A, Lombardo F, Lanzaro A, Panizzo V, Bonitta G, Ogliari C, Dell’Era A, Cavalli M, Campanelli G, Bona D (2022) Laparoscopic paraesophageal hernia repair with absorbable mesh: a systematic review. Video-assist Thorac Surg 7:26

[30] Panici Tonucci T, Asti E, Sironi A, Ferrari D, Bonavina L (2020) Safety and efficacy of crura augmentation with phasix ST mesh for large hiatal hernia: 3-year single-center experience. J Laparoendosc Adv Surg Tech A 30(4):369–372. 10.1089/lap.2019.0726 31910348 10.1089/lap.2019.0726

[31] Aiolfi A, Cavalli M, Sozzi A, Lombardo F, Lanzaro A, Panizzo V, Bonitta G, Mendogni P, Bruni PG, Campanelli G, Bona D (2022) Medium-term safety and efficacy profile of paraesophageal hernia repair with Phasix-ST® mesh: a single-institution experience. Hernia 26(1):279–286. 10.1007/s10029-021-02528-z 34716832 10.1007/s10029-021-02528-z

[32] Suppiah A, Sirimanna P, Vivian SJ, O’Donnell H, Lee G, Falk GL (2017) Temporal patterns of hiatus hernia recurrence and hiatal failure: quality of life and recurrence after revision surgery. Dis Esophagus 30(4):1–8 28375479 10.1093/dote/dow035

[33] Awais O, Luketich JD, Schuchert MJ, Morse CR, Wilson J, Gooding WE, Landreneau RJ, Pennathur A (2011) Reoperative antireflux surgery for failed fundoplication: an analysis of outcomes in 275 patients. Ann Thorac Surg 92(3):1083–1090 21802068 10.1016/j.athoracsur.2011.02.088

[34] Addo A, Carmichael D, Chan K, Broda A, Dessify B, Mekel G, Gabrielsen JD, Petrick AT, Parker DM (2023) Laparoscopic revision paraesophageal hernia repair: a 16-year experience at a single institution. Surg Endosc 37(1):624–630. 10.1007/s00464-022-09359-8 35713721 10.1007/s00464-022-09359-8

[35] Akmaz B, Hameleers A, Boerma EG, Vliegen RFA, Greve JWM, Meesters B, Stot JHM (2023) Hiatal hernia recurrences after laparoscopic surgery: exploring the optimal technique. Surg Endosc 37:4431–4442 36781470 10.1007/s00464-023-09907-w

[36] Schlottmann F, Laxague F, Angeramo CA, Sadava EE, Herbella FAM, Patti MG (2021) Outcomes of laparoscopic redo fundoplication in patients with failed antireflux surgery: a systematic review and meta-analysis. Ann Surg 274(1):78–85. 10.1097/SLA.0000000000004639 33214483 10.1097/SLA.0000000000004639

[37] Abdelmoaty WF, Dunst CM, Filicori F, Zihni AM, Davila-Bradley D, Reavis KM, Swanstrom LL, DeMeester SR (2020) Combination of surgical technique and bioresorbable mesh reinforcement of the crural repair leads to low early hernia recurrence rates with laparoscopic paraesophageal hernia repair. J Gastrointest Surg 24(7):1477–1481. 10.1007/s11605-019-04358-y 31468330 10.1007/s11605-019-04358-y

[38] Deeken CR, Chen DC, Lopez-Cano M, Martin DP, Badhwar A (2023) Fully resorbable poy-4-hydroxybutyrate (P4HB) mesh for soft tissue repair and reconstruction: a scoping review. Front Surg 12(10):1157661. 10.3389/fsurg.2023.1157661 10.3389/fsurg.2023.1157661 PMC10130450 37123542

[39] Konstantinidis H, Charisis C (2023) Surgical treatment of large and complicated hiatal hernias with the new resorbable mesh with hydrogel barrier (Phasix^TM^ST): a preliminary study. J Robot Surg 17(1):141–146. 10.1007/s11701-022-01406-9 35397107 10.1007/s11701-022-01406-9

[40] Ukegjini K, Vetter D, Dirr V, Gutschow CA (2023) Hiatus hernia repair with new-generation biosynthetic mesh: a 4-year single-center experience. Surg Endosc 37(7):5295–5302. 10.1007/s00464-023-10005-0 36976420 10.1007/s00464-023-10005-0 PMC10322952

[41] Nickel F, Muller PC, Cizmic A, Haberle F, Muller MH, Billeter A et al (2024) Evidence mapping on how to perform an optimal surgical repair of large hiatal hernias. Langenbeck’s Arch Surg 409(1):15. 10.1007/s00423-023-03190-y 10.1007/s00423-023-03190-y PMC10733223 38123861

[42] Siboni S, Asti E, Milito P, Bonitta G, Sironi A, Aiolfi A, Bonavina L (2019) Impact of laparoscopic repair of large hiatus hernia on quality of life: observational cohort study. Dig Surg 36(5):402–408. 10.1159/000490359 29925065 10.1159/000490359

[43] Wu H, Ungerleider S, Campbell M, Amundson JR, VanDruff V, Kuchta K, Hedberg HM, Ujiki MB (2023) Patient-reported outcomes in 645 patients after laparoscopic fundoplication up to 10 years. Surgery 173(3):710–717. 10.1016/j.surg.2022.07.039 36307333 10.1016/j.surg.2022.07.039

[44] Kivelä AJ, Kauppi J, Räsänen J, But A, Sintonen H, Vironen J, Kruuna O, Scheinin T (2021) Long-term health-related quality of life (HRQoL) after redo fundoplication. World J Surg 45(5):1495–1502 33502565 10.1007/s00268-021-05954-3 PMC8026436

[45] Lima DL, de Figueiredo SMP, Pereira X, Murillo FR, Sreeramoju P, Malcher F, Damani T (2023) Hiatal hernia repair with biosynthetic mesh reinforcement: a qualitative systematic review. Surg Endosc 37(10):7425–7436. 10.1007/s00464-023-10379-1 37721592 10.1007/s00464-023-10379-1

