# Supplementary material for: Does crural repair with biosynthetic mesh improve outcomes of revisional surgery for recurrent hiatal hernia?
Source: Hernia. 2024 Mar 29;28(5):1687–95. doi: 10.1007/s10029-024-03023-x (PMC11450103; doi:10.1007/s10029-024-03023-x)
Supplement: Supplementary file 1 — (DOCX 17 KB) [file 10029_2024_3023_MOESM1_ESM.docx]

**Supplementary Table 1**. Pre- and postoperative results of the SF-36 questionnaire.

|  | Mesh  (n=54) | No Mesh  (n=38) | р-value |
| --- | --- | --- | --- |
| *Physical function (PF)* |  |  |  |
| Preoperative | 53.9 (±17.7) | 46.9 (±17.9) |  |
| Postoperative | 80.7 (±19.2) | 75 (±21.7) | 0.27 |
| *Role physical (RP)* |  |  |  |
| Preoperative | 44.7 (±23.1) | 46.5 (±19.4) |  |
| Postoperative | 75.7 (±27.8) | 70,7 (±21.8) | 0.44 |
| *Bodily pain (BP)* |  |  |  |
| Preoperative | 48.7 (±9.7) | 48.1 (±9.8) |  |
| Postoperative | 79.3 (±20.3) | 73.8 (±18.8) | 0.27 |
| *General health (GH)* |  |  |  |
| Preoperative | 38.8 (±8.4) | 38.6 (±8.6) |  |
| Postoperative | 62 (±21.1) | 63.2 (±13.9) | 0.81 |
| *Vitality (VT)* |  |  |  |
| Preoperative | 41.8 (±5.3) | 41.9 (±6.7) |  |
| Postoperative | 63.4 (±17.7) | 58.3 (±15.5) | 0.22 |
| *Social function (SF)* |  |  |  |
| Preoperative | 44.1 (±7.6) | 45 (±7.8) |  |
| Postoperative | 68.0 (±18.1) | 69.2 (±19.1) | 0.78 |
| *Role emotional (RE)* |  |  |  |
| Preoperative | 36.5 (±21.1) | 44.4 (±19.9) |  |
| Postoperative | 70.7 (±28.9) | 69,8 (±25.4) | 0.89 |
| *Mental health (MH* |  |  |  |
| Preoperative | 50.2 (±6.7) | 48.5 (±6.9) |  |
| Postoperative | 71.1 (±13.2) | 68.8 (±16.9) | 0.53 |
